# Experimental Research on the Cutting of Metal Materials by Electrical Discharge Machining with Contact Breaking with Metal Band as Transfer Object

**DOI:** 10.3390/ma13225257

**Published:** 2020-11-20

**Authors:** Aurel Mihail Țîțu, Petrică Vizureanu, Ștefan Țîțu, Andrei Victor Sandu, Alina Bianca Pop, Viorel Bucur, Costel Ceocea, Alexandru Boroiu

**Affiliations:** 1 Industrial Engineering and Management Department, Faculty of Engineering, “Lucian Blaga” University of Sibiu, 10 Victoriei Street, 550024 Sibiu, Romania; viorel.bucur@ulbsibiu.ro; 2 The Academy of Romanian Scientists, 54 Splaiul Independenței, Sector 5, 050085 Bucharest, Romania; 3 Faculty of Materials Science and Engineering, Gheorghe Asachi Technical University, Blvd. D. Mangeron 71, 700050 Iasi, Romania; 4 The Oncology Institute “Prof. Dr. Ion Chiricuță” Cluj-Napoca, 34-36 Republicii Street, 400015 Cluj Napoca, Romania; stefan.titu@ymail.com; 5 Romanian Inventors Forum, Str. Sf. P. Movila 3, 700089 Iasi, Romania; 6 SC TechnoCAD SA, 72 Vasile Alecsandri Street, 430351 Baia Mare, Romania; bianca.bontiu@gmail.com; 7 Department of Marketing and Management, The Faculty of Economic Sciences, “Vasile Alecsandri” University of Bacău, 157 Mărăști Street, 600115 Bacău, Romania; costelceocea@gmail.com; 8 Automotive and Transport Department, Faculty of Mechanics and Technology, University of Pitești, 1 Târgul din Vale Street, 110040 Pitești, Romania; alexandru.boroiu@upit.ro

**Keywords:** electrical discharge machining with contact breaking, metal band, process modeling, objective functions, cutting, central composite design

## Abstract

The scientific paper presents practical research carried out by a mixed team of Romanian researchers from universities and the business environment. The research consists in applying the process of cutting metallic materials through electrical discharge machining with contact breaking using a metal band as a transfer object. The research was implemented with the help of a specially designed installation in the laboratory and subsequently all the necessary steps were taken to obtain the patent for it. Various metallic materials were cut using this process, but first of all, high alloy steels. In the global research conducted by the authors, active experimental programs and classic experimental programs were used. The composite central factorial experiment was the method that led to the most effective results in terms of interpretations and conclusions. The research as a whole includes unique elements from an engineering point of view and here we can highlight the use of a metal band as a transfer object for this type of process as well as the designed, realized, and subsequently patented installation.

## 1. Introduction

Dimensional processing through electrical discharge machining is one of the most widespread nonconventional processing processes in the world. Contact breaking electrical discharge machining is a process widely used today for cutting metal materials using mainly a disk-type transfer object. The authors of this research came to complete the range of possibilities available today using a metal band as a transfer object for processing through electrical discharge machining with contact breaking. This type of process and implicitly cutting metallic materials is mentioned in the literature by the Russian researchers Boris and Natalia Lazarenco, respectively, by the English researcher Priesley (1770). We are witnessing a continuous evolution in the use of new types of metallic materials and increasing new modern technologies in fields such as aeronautics, automotive, car construction, etc., using the so-called nonconventional technologies in which material processing is done by using and directing energies in various forms [1,2].

The literature details an important range of researchers’ concerns in order to improve the efficiency and effectiveness of processing processes in the field of nonconventional technologies [3,4]: the study of dielectrics and fluids, in particular, in the case of erosion processes, regardless of the type of process chosen [5,6,7]; complex studies and research on the processing and cutting of metallic materials and the issue of the effect of the electrode material on residual voltage [8,9]; studies on the rigidity of materials and cracks that occur in the material [10,11,12].

There are relevant analyses that have been taken into account regarding the rigidity of the processed surfaces correlated with the wear of the transfer object in order to determine the efficiency of the material sampling process depending on certain parameters such as energy source, electrical impulse duration, but also other process parameters [13,14,15].

It was found the extension of erosion processing technologies in the bio-medical field where the transfer object composition was modified by adding chromium in its component regardless of the form and type of processing [16,17,18].

It was determined that most of the contributions mentioned in the literature were found in terms of processing through electrical discharge machining with form copying [19,20,21], as well as the study of aspects worthy of consideration regarding issues of objective functions, processing productivity, volume wear, and relative wear [22,23,24]. Regarding the transfer object, specific properties of a carbon fiber reinforced polymer with a specific strength and extremely high rigidity, with a relatively low thermal expansion coefficient and a special property, namely the possibility to be able to easily model its surface, can be discussed [25,26,27]. The properties of this carbon fiber reinforced polymer are superior to other common materials.

Specific researches are presented in the literature where a redistribution of specific energies is observed in the processing of different types of materials that include aluminum alloys and sandwich panels [28,29,30]. Specific studies and research have been considered, which include issues of reliability and maintenance of material sampling processes using modern numerical simulations [31,32,33].

Through this research, it is desired to transmit a special approach regarding the modeling of technological parameters and objective functions for processing through electrical discharge machining with contact breaking using a transfer object made of a metal band, using a specially designed and subsequently patented installation and used in an industrial organization. Process parameters taken into account, together with the objective functions chosen in the context of the current process, lead to a modeling and subsequently to an optimization of the material sampling process as a whole.

## 2. General Consideration

The cutting of high steel alloys by electrical discharge machining with contact breaking (EDMCB) with electrode-tool—the metal band—represents one of the modern technological procedures of the non-conventional processing of some high steels alloy (hard and extra hard) categories, under the economic conditions of optimal efficiency [32,33]. The experimental research regarding the cutting process has emphasized, in particular, the technological aspects specific to the cutting (semi-fabricated of high steels alloy) through EDMCB, with Transfer Object (TO)—metallic band. Additionally, the existence of different values of the working parameters is highlighted, which is determined by the metal band use as a transfer object. The installations of the cutting steel process through EDMCB, has in its structure a tool—TO—in a metal disk form (this is the most commonly applied constructive solution), a solution subject to specific restrictions determined by the dimensions and a large size of semi-finished products. The replacement of the metal disk with the metal band, as a tool—TO—to carrying out the cutting operations, fundamentally changes the constructive solutions applied so far, determining completely new construction forms for the new installations. This replacement influences the range of phenomenological, constructive, and technological constraints, which limit the using possibilities of the metal disc, as a tool (determined by the range of the semi-finished products) in the specific processes of the metallurgical industry in particular. The multiple research options applied, led to the identification of optimized values of the construction elements of the metal band (length, width, thickness), as well as for the ends of the connection, to form a closed contour, strong and durable. At the same time, the correlation of the diameter of the flywheels was considered, with the distance between their axes and related to the elements in the structure of the experimental installation/experimental stand. The variables measurement of the working parameters used and presented in the paper is measured with standardized measurement and control gages used on a large scale. For example *Ra*—which is the arithmetical mean deviation of the assessed profile—was determined with the Surtronic S-116 Series Surface Roughness Tester (Taylor-Hobson, Warrenville, IL, USA). The measurement capability of this gauge has the range of 200 [µm] and the resolution of 100 [nm]. The stylus tip radius is 5 µm. It is able to calibrate to ISO 4287 roughness standard. The filter cut-off is 0.25 mm and the filter type is 2CR/Gaussian. The evaluation length is suitable for 0.25–17.5 mm, with a measuring speed of 1 mm/sec. During the experimental research, the cutting time was measured with the chronometer. The cutting was measured with the feller gauge and the thermal influenced zone was analyzed with Micro-Vu VERTEX 261 (Micro-Vu, Conde Lane Winsdor, CA, USA).

## 3. The Experimental Research Development

For the experimental research development under optimal conditions, the following aspects have been considered:

(a) ensuring the material conditions regarding the establishment of the processing objects of high steel alloys category, under the form of rolled steel:
  - 34 MoCrNi15–ϕ 40 mm;
  - RUL–1–ϕ 26 mm.
(b) the work parameters observed during the cutting process:
  - mechanical;
  - the metal band thickness (*s*) [mm];
  - the relative speed (*v_r_*) [m/s];
  - the feed rate (*v_a_*) [m/min].
(c) electrical:
  - the working current (*I*), [A];
  - discharge voltage (*U*), [V];
  - polarity (*+,−*).
(d) technological:
  - the cutting time (*t_t_*), [s];
  - the cutting width (*l_t_*), [mm];
  - the arithmetical mean deviation of the assessed profile (*R_a_*), [µm];
  - the thermal influenced zone (*TIZ*) [mm].
(e) validation conditions.

The conditions that need to be fulfilled by the whole parameters considered as state variables have been complied with the following:

- the direct measuring possibility;
- the lowest dispersion of the values during the experiments.

The analysis of the main categories of parameters (mechanical, electrical, technological) was performed using a mathematical modeling program within the research program [34,35,36].

### 3.1. Main Parameters Considered as State Variables

The research done on the experimental installation specifically aimed to some technological aspects related directly to the cutting by electrical discharge machining with contact breaking with electrode-tool—the metal band.

The article presents some of the obtained results within the “Research Program” after having analyzed the main parameters of the categories considered stating the following variables:

- Cutting time (*t_t_*), [s];
- Cutting width (*l_t_*), [mm];
- Arithmetical mean deviation of the assessed profile (*R_a_*), [μm];
- Thermal influenced zone (*TIZ*), [mm].

As independent functions, considered as input measures, the following were taken into consideration:

- The working current (*I*), [A];
- The feed rate (*v_a_*) of the Processing Object (PO), [m/min];
- The relative speed of the electrode-tool (*v_r_*) of the TO, [m/s];
- The metal band thickness (*s*), [mm].

The influence of each work parameter (or factor) grouped was observed, while the others were maintained constant.

### 3.2. Drawing Dependency Diagrams for State Variables

**Figure 1:**
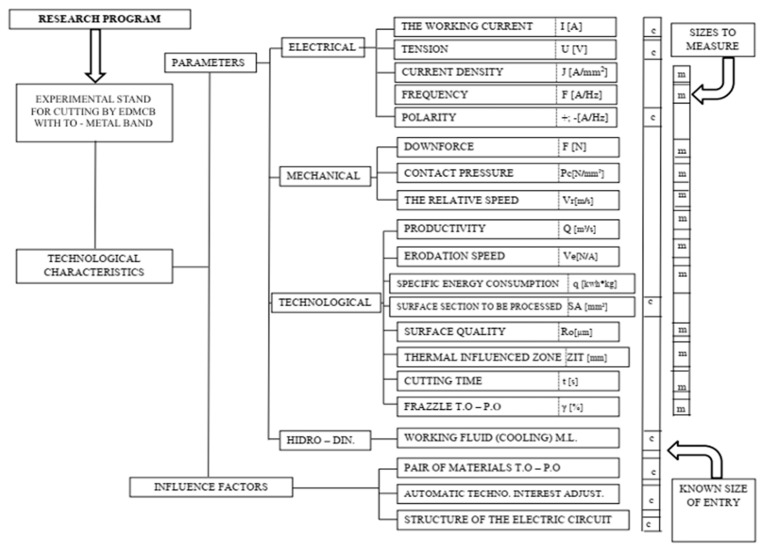
The experimental research programming through processing by electrical discharge machining with contact breaking (EDMCB) with Transfer Object (TO)—metal band.

Performing the experimental research in the processing/cutting by electrical discharge machining with contact breaking (EDMCB) with TO—metal band—according to the Research Program in Figure 1, ensured the succession of the experimental determination of the concrete values of some parameter categories (electrical, mechanical, and technological).

The dependency diagrams of the state variables are presented below, considering the values mentioned in Table 1 and Table 2, for the two categories of steel (34 MoCrNi15-φ 40 mm and RUL-1-φ 26 mm).

#### 3.2.1. For 34 MoCrNi15-φ 40 mm

**Figure 2:**
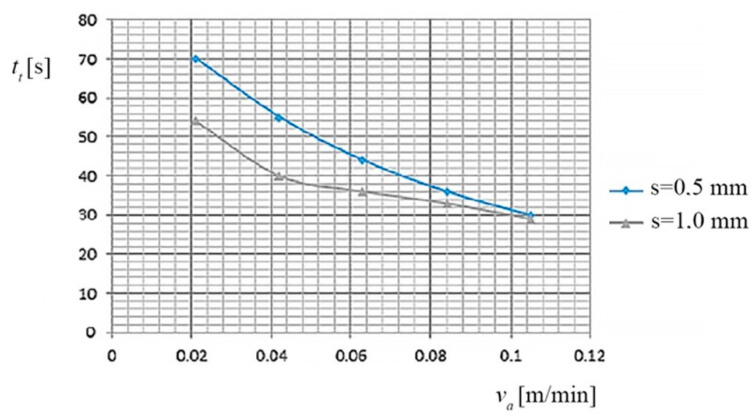
Dependence of the *t_t_*, on *v_a_* of the Processing Object (PO) and on the thickness (*s*) of the metal band.

**Figure 3:**
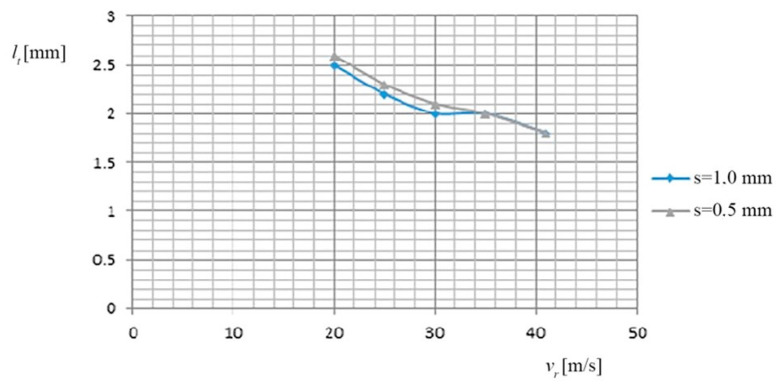
Dependence *l_t_*, on the *v_r_* of TO and on the thickness (*s*) of the metal band.

**Figure 4:**
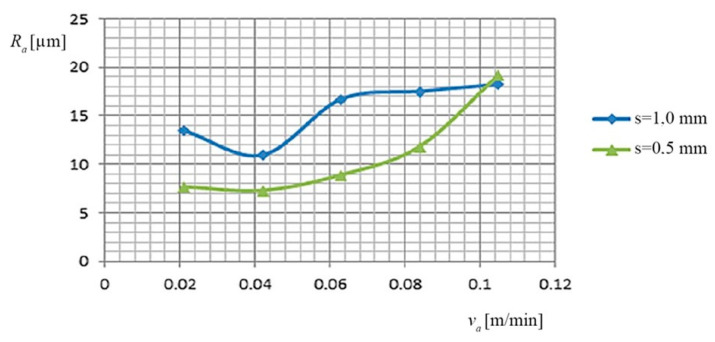
Dependence on the *R_a_* by *v_a_* of the PO and the thickness of the metal band (*s*).

**Figure 5:**
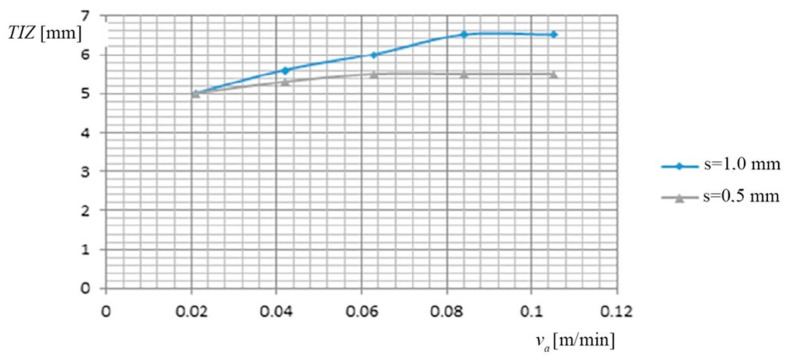
Dependence Thermal Influenced Zone (*TIZ*) on PO and thickness (*s*).

(a) The dependence of the cutting time (*t_t_*), the feed rate (*v_a_*) of the PO, and the thickness of the metal band (*s*), is shown in Figure 2, based on the data presented in Table 3.

(b) The dependence of the cutting width (*l_t_*), on the relative speed (*v_r_*) of the TO and on the thickness of the metal band (*s*), is shown in Figure 3, based on the data in Table 4.

(c) The dependence of the *R_a_*, on the working current (*I*), on the thickness of the metal band (***s***), and on the *v_a_* of PO, is shown in Figure 4, based on the data presented in Table 5.

(d) Dependence of the thermal influenced zone (*TIZ*), on the working current (*I*), on the feed rate *v_a_* of PO, and by the thickness of the metal band (*s*), is shown in Figure 5, based on the data presented in Table 6.

#### 3.2.2. For RUL-1-φ 26 mm

**Figure 6:**
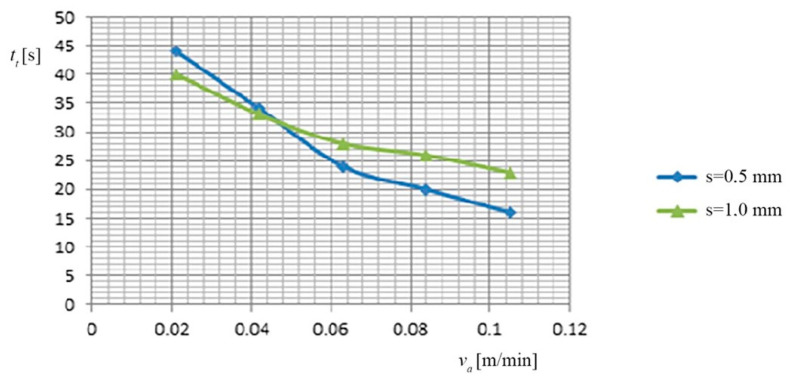
Dependence on the cutting time (*t_t_*), the feed rate (*v_a_*) of the PO, and the thickness of the metal band (*s*).

**Figure 7:**
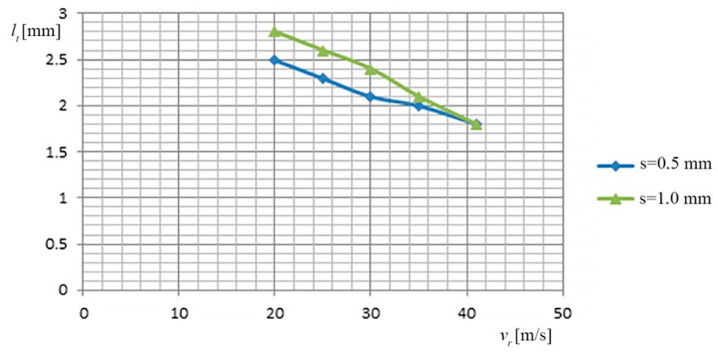
Dependence of the cut width (*l_t_*) of the relative speed (*v_r_*) and the thickness of the metal band TO (*s*).

**Figure 8:**
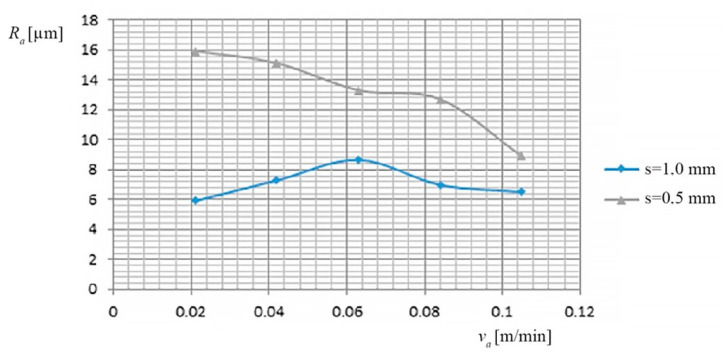
Dependence on the *R_a_*, the feed rate of the PO and the thickness of the metal band.

**Figure 9:**
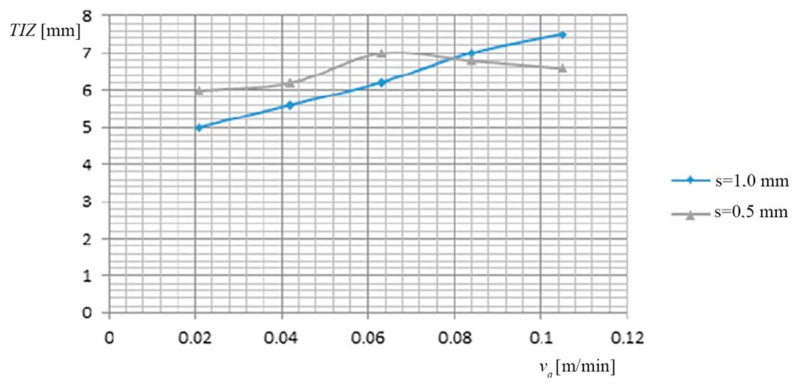
Dependence *TIZ* on the feed rate *v_a_* of PO and on the thickness of the metal band (*s*).

(a) The dependence of the cutting time (*t_t_*), the feed rate (*v_a_*) of the PO, and the thickness of the metal band (*s*), is shown in Figure 6, based on the data presented in Table 7.

(b) The dependence of the cutting width (*l_t_*) on the relative speed (*v_r_*) of the TO and on the thickness of the metal band (*s*), is shown in Figure 7, based on the data of Table 8.

(c) The dependence of the *R_a_*, on the feed rate *v_a_* of PO and on the thickness of the metal band (*s*), is shown in Figure 8, based on the data of Table 9.

(d) The dependence of the thermal influenced zone (*TIZ*), on the feed rate *v_a_* of PO and on the thickness of the metal band (*s*), is shown in Figure 9, based on the data presented in Table 10.

### 3.3. Interpretation of the Dependency Graphs Charts

(a) The research highlights the following aspects:

- with the increasing speed of the PO, the cutting time decreases;
- when increasing the feed rate of PO by 0.021 [m/min] to 0.084 [m/min], the cut-off time is reduced to 55%, reflecting a significant processing productivity increase (as a result of the increase *v_a_* of PO);
- for the metal band thicknesses (*s* = 1.0 mm), the range of the PO feed rate was limited to 5 levels (avoiding the breaking of the metal band at higher speeds);
- for increasing the cutting productivity, it is recommended to use a cutting time (*t_t_*) correlated to the pressure between TO and PO, metal band thicknesses of *s* = 0.5 mm, and values of (*v_a_*) of PO greater than 0.063 m/min.

(b) In this case, minimum deviations were observed for the 2 categories of materials cut:

- at the relative speeds of 20–25 [m/s] of the TO, due to the “extension” (during operation) of the metal band, its “sweeps and motions” appear, which cause an intensification of the “wrong springs” (lateral), between the TO and the PO, which causes an increase in the cutting width;
- the cutting width (*l_t_*) has the optimum values (≈2 mm) at high relative speeds (41 m/s) and average values of the working current (7500 A), when the metal band greatly reduces its lateral play (sweeping tendency).

(c) The variation mode of the *R_a_* according to the working regime (*I*, *v_a_*, *v_r_*, *s*) was followed.

- low values of *R_a_* were observed at the *v_a_* of PO, in the range of 0.042–0.084 m/min, and to the working current values between 600–1000 A;
- the thickness of the metal band (*s*) causes high, different roughness for small values of the working current, between 300 and 600A.

(d) The cutting of the two categories of materials (34 MoCrNi15-φ 40 mm and RUL-1-φ 26 mm), under different regimes and with the variation of the cooling agent, showed different *TIZ*, these being influenced by the other parameters (the working current, the feed rate of the PO, and the thickness (*s*) of the metal band).

- the cooling agent (technological water) was “ordered” to ensure the cooling of the work area;
- *TIZ* has maximum values, in the exit zone of the metal band from the material, due to the eroded metal, reached the melting state and due to the insufficient cooling, the main cause being both the working current and the advance speed of the PO. These frequently encountered situations were observed primarily visually, and then following the repeated experiments, it can be concluded the important main cause which “is due to the working current speed but also the feed rate of the object to be processed”.

The thermally influenced zone has the maximum value in the output area of the metal band from the material, due to eroded metal so that it reaches the molten state and then cooled but insufficiently. Based on the authors experience in this field, it can be considered that the main cause is due to the working current but also to the feed rate of the processing object.

**Figure 10:**
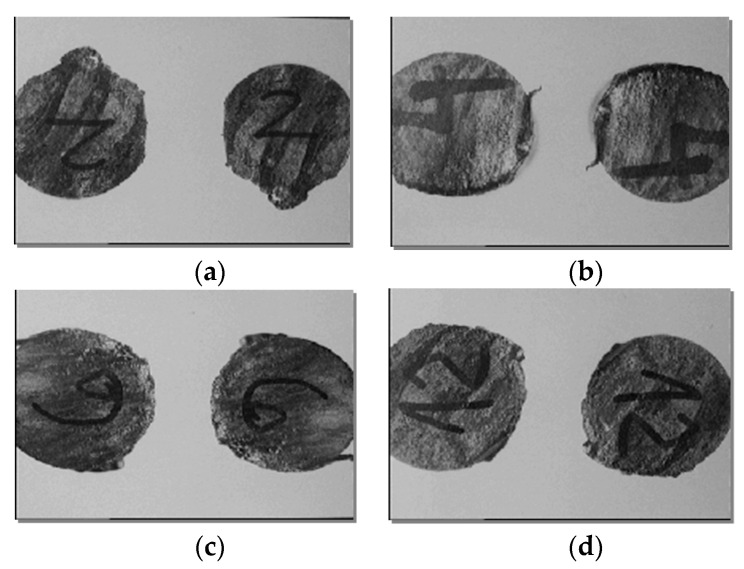
Dependence *TIZ* on the feed rate *v_a_* of PO and on the thickness of the metal band (*s*): (**a**) Sample 1, (**b**) Sample 2, (**c**) Sample 3, (**d**) Sample 4.

The different cutting regimes for the processed samples determined different influences on the main parameters pursued in the experimental research (*t_t_*, *l_t_*, *R_a_*, and *TIZ*). In Figure 10, it is presented the macrostructural analysis of 4 samples which were cut with metal band by EDMCB with different regimes.

The cut surfaces show specific cutting traces of EDMCB:

- different structures and sizes of *TIZ*, due to uneven cooling with coolant during the surface cutting process;
- areas of molten and re-solidified material (characteristic of the Joule–Lenz effect), especially in the exit part of the metal band from the PO (Figure 10a,c);
- the quality of the cut is not the same on the whole cut area;
- to the pressure forces higher than 400 N, the number of contacts between TO-PO increases, the metal band tends to “sweep” changing the perpendicularity and causing an accentuation of “erroneous” arc, between it and PO and a decrease of the quality of the cut surface.
- the extension of the metal band determines the amplification of the discharges in non-stationary arc, between it and the PO having as an effect the deterioration of the respective surfaces (Figure 10b,d).

## 4. Experimental Installation/Stand, for the Study of the Processing by EDMCB, With TO—Metallic Band, Of the High Steels Alloy (Hard and Extruded)

In order to establish the technological characteristics, the correlation between the factors and the parameters, an experimental installation/stand was designed and executed for the cutting of high steels alloy, considering the functions and the structure resulting by the dimensional analysis and the research results presented in the specific literature.

The experimental installation/stand allowed a wide limit of variation of some technological parameters and of their measurement, (this being designed and structured based on the idea diagrams and the morphological matrices).

NOTE: The analysis and realization of the structure of the experimental installation took into consideration the influence of some parameters and factors specific to the processing by EDMCB (some of them are also found in the machines that use as TO—the metal disk).

### 4.1. The Main Components of the Experimental Installation’s Structure

The experimental installation is made out of two main subassemblies:

- The electricity supply source;
- The experimental cutting stand.

(a) The electricity supply source:

- Electric supply source 1000A, [A].

It has the role of generating the erosive agent, bearing the technical characteristics, mentioned in the “Power source book”, being connected to a 380 V industrial network, complying with the norms of protection against electrocution.

- Electric command board;
- The control of the feeding systems (of the experimental stand), made with the help of a thyristor bridge that includes:
  ○ electric motor;
  ○ reducer;
  ○ screw.

(b) The experimental cutting stand:

It is made out of a metal frame (frame—welded construction) which gives it an optimal behavior with dynamic shocks and vibrations.

**Figure 11:**
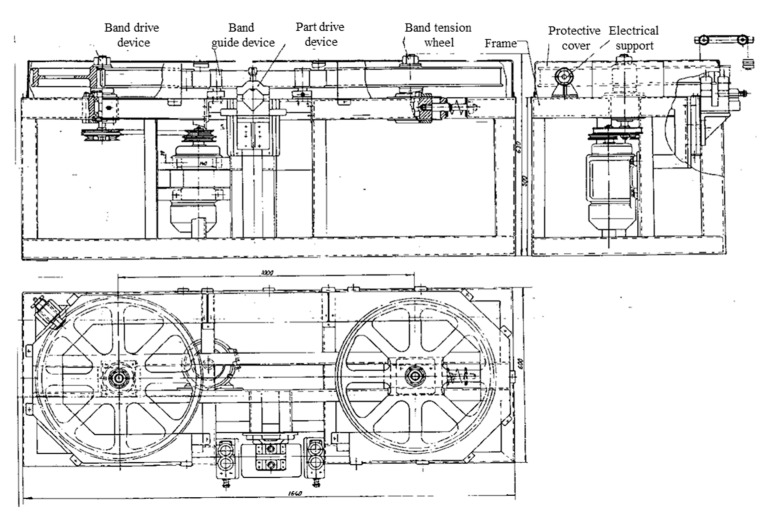
The experimental stand assembly.

The experimental stand scheme is presented in Figure 11.

### 4.2. The Main Elements Ensuring the Main Functions

The systems which ensure the realization of the main functions (engaging, lengthening of the metal band, guiding, etc.) are mounted on the frame. Additionally, mounted are:

- The mechanism of the processing objects;
- The orientation guides of the electrode-tool;
- The positioning and advance subassembly of the processing objects;
- The receiver subassembly of the working current;
- The cooling and evacuation subassembly of the eroded material and many others.

The parts of the experimental installation in the working area are isolated and covered by protection elements for the benefit of the operating workers which observe it.

The present installation is a part of the field of non-conventional processing and has as an object a cutting installation by electrical discharge machining with contact breaking, with metal band transfer object. The installation can be applied in the machine building industry, in the metallurgical industry, in foundries, etc., being indicated, in particular, for cutting parts of materials difficult to process by cutting, which have a high and very high hardness, or which are resistant to corrosion, and cavitation, refractory materials, brittle materials, etc. The installation can also be used to cut the supply masses and casting networks of some castings. This plant, although clearly superior to conventional cutting machines, still has the main drawback of the high cost of processing due to high electricity consumption. This high consumption of electricity is mainly generated by energy losses due to the lateral electrical discharges, produced between the front faces of the electrode disc used as an object of electricity transfer and the cutting surfaces already made in the processed object, discharges which no longer contribute to the actual cutting process. As the cutting progresses, the areas of the contact surfaces between which these parasitic side discharges occur also increase, having as a consequence a certain instability of the electrical discharge machining process as well as a relatively low productivity.

The technical problem solved by the installation consists of the reducing of the contact surfaces areas between the electricity transfer object and the processed object and thus the accentuated decrease of the energy losses by the lateral electric discharges produced between the two objects. The contact erosion cutting plant with contact breaking, with metal band transfer object, solves the mentioned technical problem and eliminates the above disadvantages. That consists of an endless metal band with the role of electricity transfer object, positioned vertically and performing a rectilinear, forward, and interacting motion, with a processing object arranged tangentially under the metal band. This is wound on two flywheels with vertical axes, of which a drive flywheel set in rotation by an electric motor and a belt drive. The second flywheel is a driven flywheel, supported by a movable bearing coupled to a metal belt tensioner which is also held firmly in a vertical plane, by means of at least two identical guiding devices, placed on either side of the workpiece caught in a fastening and advancing device which, moving on a guide plate, under the action of suspended counterweights at the end of a steel cable, performs the advance movement of the object to be processed, perpendicular to the trajectory of the metal band and the contact pressure necessary to carry out the process of electrical discharge machining, with contact breaking, supply electrical energy of the processing process being made by direct connection of the object to be processed to one pole of the power supply, the other pole being connected to the metal band, by means of several current receiving devices, arranged on both sides of the metal band, both on one side and on the other side of the object to be processed.

The endless metal band, with the role of transfer object, is made of narrow carbon steel band, cold rolled, having 30–50 mm width and 0.3–1.00 mm thickness. The drive flywheel and the driven flywheel are provided at their periphery with a groove for laying and guiding the metal band.

The tensioning device of the metal band consists of a stretching axis supported and guided axially by a plate-bearing integral with the frame and provided with a threaded area screwed into a fixed plate, stiffened with the bearing plate by some connecting columns, the tensioning shaft being coupled by an elastic ring to a bearing plate which supports the movable bearing of the driven flywheel. A metal band guide device consists of two guide rollers, with parallel axes, through which passes the metal band, the guide rollers rotating freely on a fixed axis and, respectively, on a movable axis, the fixed axis being mounted on a guide body in which a sledge can be supported which supports the movable shaft of the guide roller provided at the ends with sills and which, together with the sledge, can approach or move away from the guide roller with fixed shaft by manually driving. The device for fixing and advancing the object to be machined consists of an upper body, fixed to the end of the steel cable with counterweights and provided with screws for fixing the object to be machined on a prism fixed on a bracket that can move on a plate with guides, fixed on the frame, under the action of counterweights suspended on the steel cable. A current receiving device consists of several collecting brushes pressed on the side of the metal band by the force of compression springs, mounted together with the collecting brushes in a brush holder box made of insulating material, fixed on a support provided with some oval holes through which some fixing screws pass on an element of the frame.

## 5. Using the Central Composite Factorial Experiment When Processing the Results

The experimental results obtained have been processed with the help of a Program Package which made it possible to analyze, model, optimize, and statistically interpret the data. Thus, the data obtained (namely the equations which express the mathematical form of the objective functions, as well as the values of the regression coefficients) have been statistically modeled, thus making the modeling and optimization of the processing through electrical discharge machining with contact breaking with electrode-tool—metal band.

### 5.1. Interpreting the Graphs of the State Variables According to the Estimated Values and the Measured Ones

**Figure 12:**
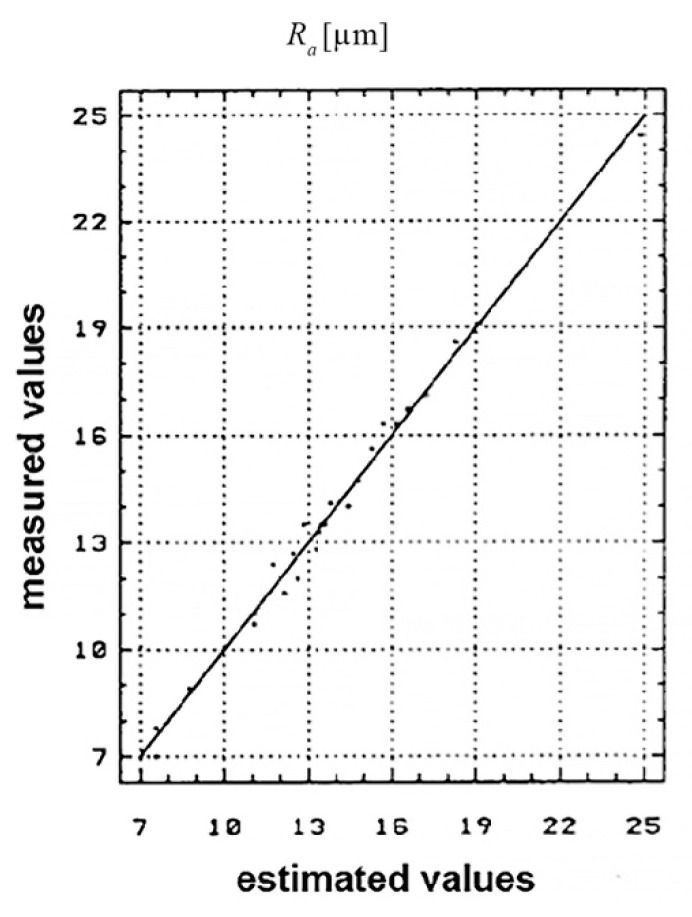
*R_a_* measured values [μm].

**Figure 13:**
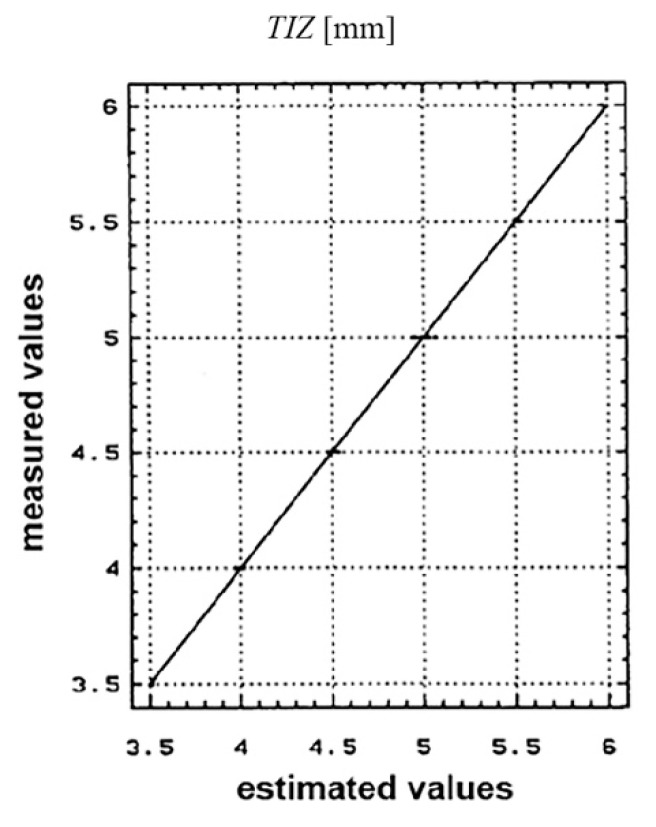
Thermal influenced zone values measured [mm].

For the four state variables (*R_a_*, *TIZ*), the graphs of the estimated values and the values measured during the experimental research are presented in Figure 12 and Figure 13.

The *R_a_* distribution of the estimated and measured values is shown in Figure 12.

The *TIZ* distribution of the estimated and measured values is shown in Figure 13.

The plan representation of the results interpretation was done using the correlation diagrams. It was seen that the ratio between the values measured and the estimated ones within the conducted research, is found within the K E [0.85, 1] limits; which lead to the conclusion that the values obtained experimentally are viable and reasonable. This aspect was also highlighted by the spreading manner of the experimental results obtained.

### 5.2. Interpreting the PARETO Diagrams

**Figure 14:**
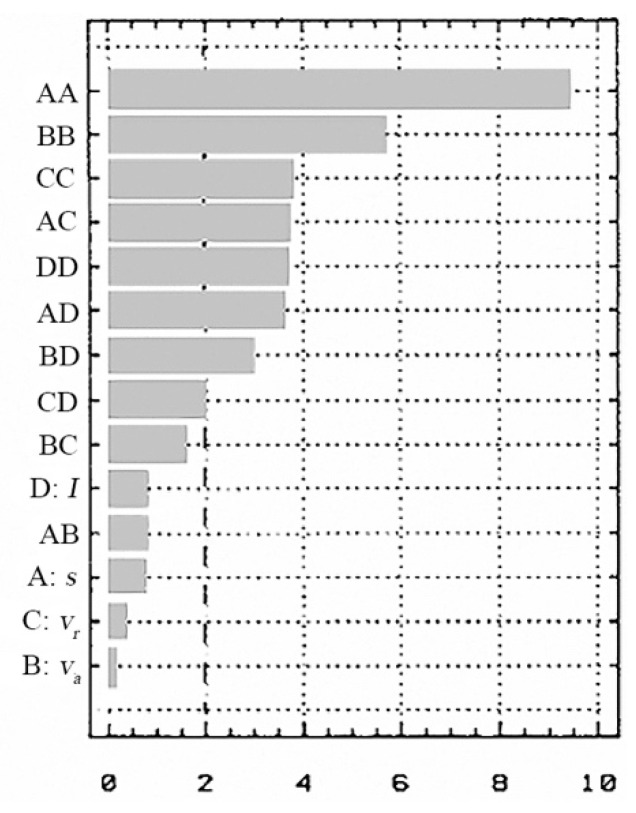
The input factors influence on *t_t_* [s].

**Figure 15:**
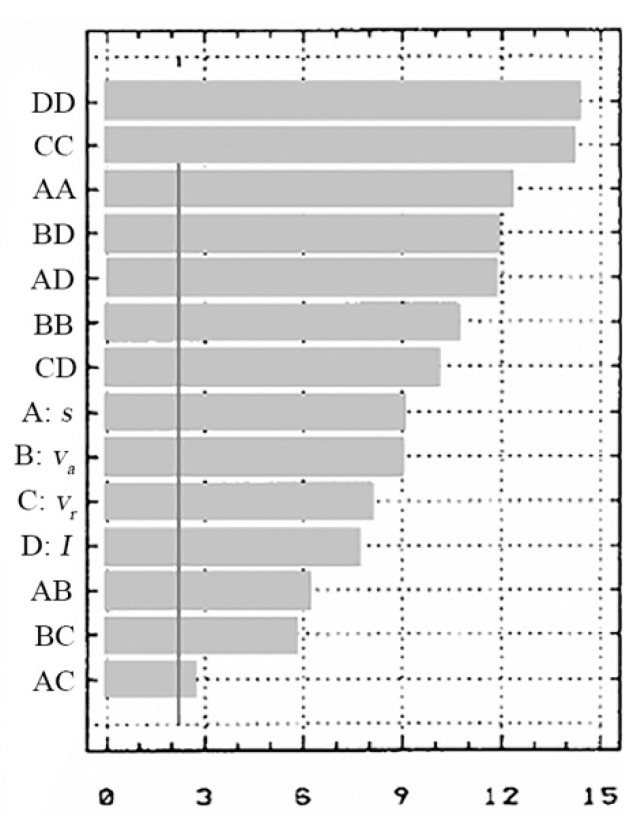
The input factors influence on *R_a_* [μm].

For the state variables (*t_t_*, *R_a_*), in Figure 14 and Figure 15, the standard effects are presented according to the influence of the input factors (*v_a_*, *v_r_*, *s* and *I*) and the chosen performance criteria.

It is found that the influence of the independent factors in the process depends on the performance criterion chosen.

In the influence values for the objective functions case–will be highlighted which of the input parameters (*I*, *s*, *v_r_* and *v_a_*) have the biggest influence on these functions.

(a) For the cutting time (*t_t_*) the biggest influence is the working current (*I*), followed by the thickness of the metal band (*s*) and the relative speed (*v_r_*) of the electrode-tool;

(b) For the cutting width (*l_t_*) the biggest influence is the feed rate (*v_a_*) of the processing objects followed by the working current (*I*) and thickness of the metal band (*s*);

(c) For the *R_a_* the most important parameter is the metal band thickness (*s*), followed by the feed rate (*v_a_*) of the processing objects and the relative speed (*v_r_*) of the working current (*I*);

(d) For the thermal influenced zone (*TIZ*) the biggest influence is the processing objects feed rate (*v_a_*), followed by the working current (*I*) and the TO relative speed (to a lower degree).

The standard effects (the PARETO diagrams) give an overall view of the evolution and variation of the process’s independent variable influences, according to the chosen and analyzed criteria.

In these PARETO diagrams, the terms coefficients which constitute the objective functions (*t_t_*-A, *l_t_*-B, *R_a_*-C, *TIZ*-D) are represented by the markings: AA, BB, CC, DD, AB, AC, AD, BD, BC and CD.

### 5.3. Interpreting the Sections Through the Variation Graphs of the Objective Functions for Each State Variable According to the Determined Input Parameters

The analysis of the diagrams and sections of the state variables related to the dependent ones (considered as input measures) will be presented next for each of them.

#### 5.3.1. For the Cutting Time (*t_t_*)

**Figure 16:**
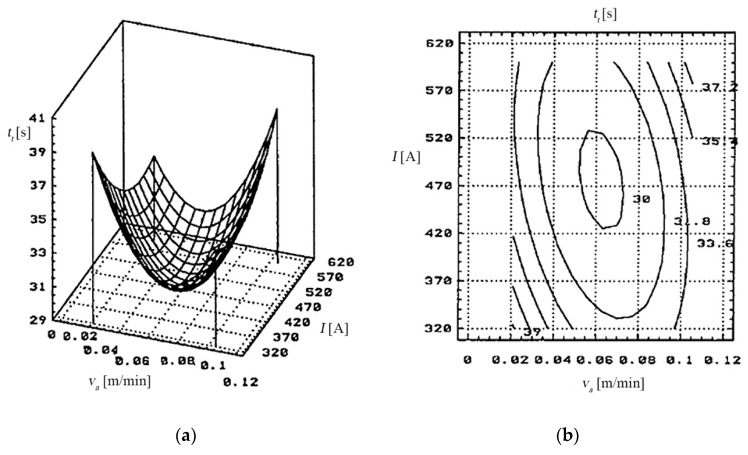
The dependency of the cutting time (*t_t_*) to the feeding rate (*v_a_*) and the working current: (**a**) 3D graph, (**b**) 2D graph.

- The dependency of the cutting time (*t_t_*) to the feeding rate (*v_a_*) of the processing object and the working current (*I*) is presented in Figure 16.
- Thus, the cutting time (*t_t_*) reaches the optimal values (30–32) [s] when the input parameters within the working cycle have been set for the following values:
  *v_a_*—between 0.042–0.084 [m/min];
  *I*—between 450–550 [A];
  *s*—between 0.5–0.8 [mm].
- The dependency of the cutting time (t_t_) to the relative speed (*v_r_*) of the electrode-tool and the working current (*I*).

The cutting time (*t_t_*) reaches the optimal values (30–32) [s] when the input parameters are set for the following values:

*v_r_*—between 30–35 [m/s];
*I*—between 450–550 [A];
*s*—between 0.5–0.8 [mm].

Each of the input parameters determines a certain influence on the cutting time (*t_t_*).

#### 5.3.2. For the cutting width (*l_t_*)

The dependency of the cutting width (*l_t_*) to the relative speed of the electrode-tool and the working current (*I*).

The length of the cutting found at values between 1.8–2.2 mm was obtained with the following values of the input measures:

- the working current: *I* between 550–600 [A];
- the relative speed of the electrode-tool *v_r_* > 40 [m/s];
- the metal band thickness: *s* between 0.6–0.8 [mm].

During the experiments the cutting’s width variation (*l_t_*) according to the work parameters and the work cycle but not necessarily in a pre-established dependency, was noted.

**Figure 17:**
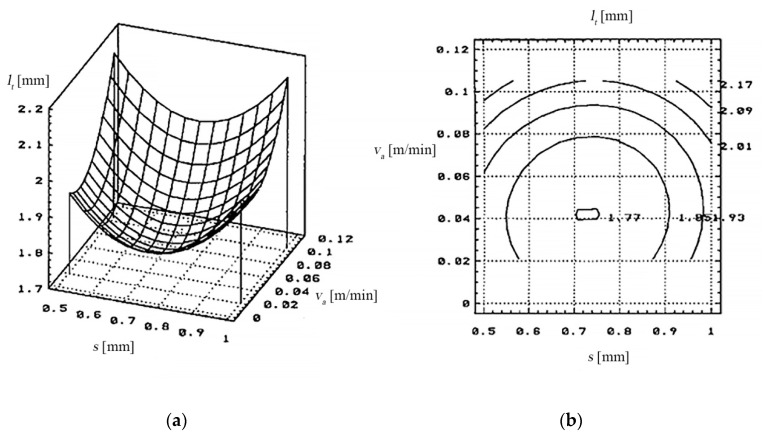
The dependency of the cutting width (*l_t_*) to the thickness (*s*) of the metal band and the feeding rate (*v_a_*) of the processing object: (**a**) 3D graph; (**b**) 2D graph.

The dependency of the cutting’s width (*l_t_*) to the metal band thickness (*s*) and the feeding rate (*v_a_*) of the processing object is presented in Figure 17.

Variations of the cutting width between 1.8–2.2 mm were obtained with the following values of the input variables:

- the working current: *I* between 400–500 [A];
- the feed rate of the processing objects: *v_a_* between 0.021–0.042 [m/min];
- the metal band thickness: *s* between 0.5–0.7 [mm].

#### 5.3.3. For the Arithmetical Mean Deviation of the Assessed Profile (R_a_)

**Figure 18:**
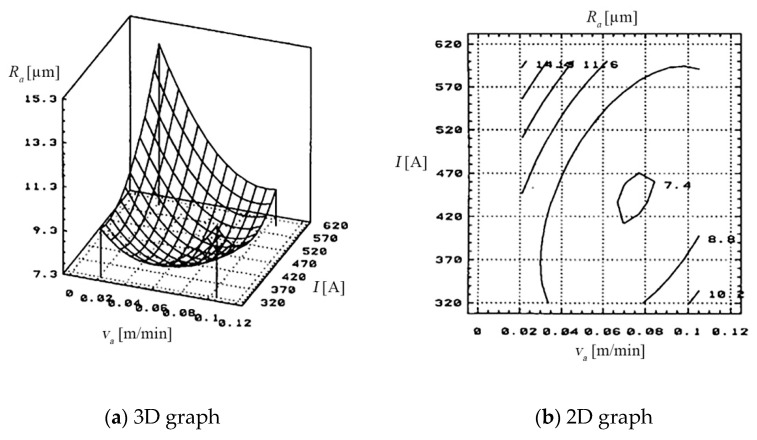
The dependency of the arithmetical mean deviation of the assessed profile (*R_a_*) to the feeding rate (*v_a_*) of the processing object and the working current (*I*): (**a**) 3D graph, (**b**) 2D graph.

The surface quality according to the feed rate (*v_a_*) of the processing object and the working current (*I*) is presented in Figure 18.

The optimal values of the rigidness *R_a_* = 9.5 [µm ] were obtained the following values of the input measures:

- the feed rate: *v_a_* between 0.042–0.063 [m/min];
- the working current: *I* between 400–500 [A].

The quality of the cut surface was influenced by the working cycle and the agent for evacuating eroded products.

**Figure 19:**
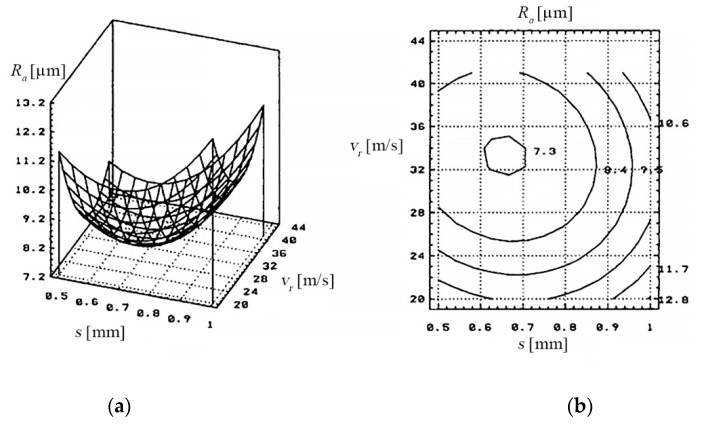
The dependency of the arithmetical mean deviation of the assessed profile (*R_a_*) to the thickness (*s*) of the metal band and the relative speed (*v_r_*) of the electrode-tool; (**a**) 3D graph, (**b**) 2D graph.

The dependency of the arithmetical mean deviation of the assessed profile (*R_a_*) to the metal band thickness (*s*) and the relative speed (*v_r_*) of the electrode-tool is presented in Figure 19.

The *R_a_* obtained after cutting, comprises a wide range of values (5–20 [µm] for *R_a_*, determined by the working regime (hard/soft) and by the cooling and evacuation agent of erosive products. The cooling agent of erosive products (technological water), is essential to obtain a quality of the surfaces cut as best as possible.

Overheating and slow evacuation of erosion products, caused by insufficient cooling agent and improper cooling, cause deterioration of the cut surface.

#### 5.3.4. For the Thermal Influenced Zone

**Figure 20:**
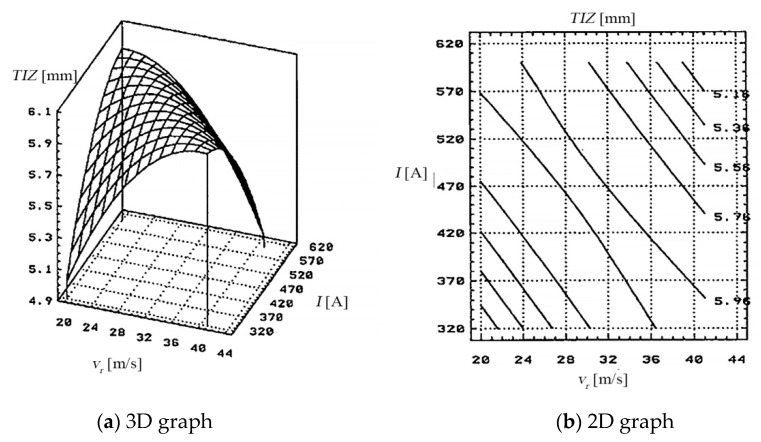
The dependency of the thermal influenced zone (*TIZ*) to the relative speed (*v_r_*) of the transfer object and the working current (*I*): (**a**) 3D graph, (**b**) 2D graph.

The dependency of the thermal influenced zone (*TIZ*) to the relative speed (*v_r_*) of the electrode-tool and the working current (*I*) is presented in Figure 20.

The thermal influenced zone had a different manifestation after the experimental tests were done, due to the impact of the working cycle (hard/soft) and the cooling agent applied.

Figure 20 highlights the dependency of the thermal influenced zone, whose values surpass 6 [cm] at a “hard” working cycle, for values of the working current between 500–620 [A] and relative speeds of the electrode-tool between 24–36 [m/s].

From the thermal influenced zone, variation graphs analysis, and of the sections using graphs, it is shown that the working parameters have been set at these values:

- the feed rate of the processing objects: *v_a_* between 0.020–0.042 [m/min];
- the relative speed: *v_r_* between 20–30 [m/s];
- the working current: *I* between 400–500 [A];
- the metal band thickness: *s* between 0.5–1 [mm].

After using these working parameters, thermal influenced zone with values smaller than 5 [cm] were obtained.

## 6. Results and Discussions

The scientific research has a wide applicability in the industry. The used stand was patented and subsequently optimized structurally and functionally, which led to its manufacture in small series to be used in the industry. The use of this equipment can lead to the assurance of optimal process parameters when cutting through electrical discharge machining with contact breaking with metal band as the transfer object.

The research conducted on the obtained results with the Program Package (Factorial Programs help) has allowed emphasis to be placed on state variables (*t_t_*, *l_t_*, *R_a_* and *TIZ*) of some of the values which, statistically interpreted, lead to substantial conclusions and motivations.

The experimental installation which considered the structure and the functions resulted from the dimensional analysis allowed the realization of the work process, ensuring multiple possibilities of technological research.

The experimental results obtained have been processed using a Statistic Program (“Statistic Data System 2000” Program Package) which made it possible to analyze, model, optimize and statistically interpret this data. Thus, the obtained data (namely the equations which express the mathematical form of the objective functions, as well as the values of the regression coefficients) have been statistically modeled, thus showing the modelling and optimization of the processing through electrical discharge machining with contact breaking with electrode-tool—metal band.

By interpreting the graphs of the state variables according to both the estimated values and the measured ones, it was seen that the ratio between the values measured and the ones estimated by the conducted research is found within the K E [0.85, 1] limits. This leads to the conclusion that the values obtained experimentally are viable and reasonable; this aspect also being highlighted by the spreading manner of the experimental results obtained.

By interpreting the Pareto diagram, it shows which of the input parameters (*I*, *s*, *v_r_* and *v_a_*) have the biggest influence on the objective functions:

- For the cutting time (*t_t_*)—the biggest influence is the working current (*I*);
- For the cutting width (*l_t_*)—the feed rate (*v_a_*) of the processing objects;
- For the arithmetical mean deviation of the assessed profile (*R_a_*)—the most important parameter is the metal band thickness (*s*);
- For the thermal influenced zone *TIZ*—the biggest influence is the feed rate (*v_a_*) of the processing objects;

By interpreting the sections through the variation graphs of the objective functions for each state variable according to the determined input parameters, it is seen that:

- For the cutting time (*t_t_*)—each of the input parameters determines a certain influence on the cutting time (*t_t_*).
- During the experiments, it was noted that the variation of the cuttings width (*l_t_*)—according to the work parameters and the work cycle, is not necessarily in a pre-established dependency.
- The quality of the cut surface was influenced by the working cycle and the agent for evacuating eroded products.
- The values of the rigidness have been influenced by overheating due to the inadequate cooling and insufficiency of the cooling agent, evacuation and slow evacuation of the eroded products.
- The thermal influenced zone had a different manifestation after the experimental tests were done, due to the influence of the working cycle (hard/soft) and the cooling agent applied.

For specialists in the field, understanding these interpretations may constitute a starting point in implementing this performing, efficiency cutting procedure.

## 7. Conclusions

Economic efficiency is an essential criterion that allows the establishment of cutting technology that provides the greatest economic effects, essentially maximizing the effect/effort ratio.

The experimental results highlight the economic advantages obtained when cutting semi-finished products of alloy steels, by EDMCB, using as TO (tool), the metal band.

Following the experiments performed and the results obtained, it became possible to apply a new technology for cutting alloy steels, which can successfully replace existing technologies, specific to conventional fields (by cutting, electric arc, thermal energy, etc.).

Characteristic for the new cutting technology by EDMCB developed are:

- increasing the productivity of social work, respectively saving social work;
- improving the quality of the cut areas;
- facilitating work and changing its character.

The present scientific paper through its wide approach of the problems related to the cutting by electrical discharge machining with contact breaking using as a transfer object the metal band, has proved that it is one of the modern technologies that can solve some problems related to the processing materials with special characteristics (hard, extra hard, etc.) in conditions of adequate technical and economic efficiency. The analysis and research carried out during the elaboration of this scientific paper in the field of non-conventional processing (of cutting materials by EDMCB) have highlighted the performances and advantages offered by this process. The research activity carried out led to the highlighting of some technological and managerial aspects related to the cutting of some alloy steel materials, using as a metal object of transfer, the metal band, and the completion of the established research directions allowed to make important original contributions.

The obtained results in the theoretical and experimental research, as well as the original contributions made during the practical experiments performed with the presented installation (State Patent Office for Invention and Trademarks no. 122346) allowed the elucidation of technological and managerial aspects considered by the authors. Even if in the approached field there could be other things left to research and elucidate, the authors express their conviction that the elaborated scientific paper includes elements of novelty and solutions, tangible results presented in qualitative and quantitative form, future trends and clearly explained reasons, which applied in industry, will directly lead to the elimination of problems of a technological nature that still persist today, as well as to outstanding decisions and results.

The arguments underlying the above statements take into account the calculation of the optimal manufacturing batch, the economic calculation justifying the adoption of the proposed variant of EDMCB cutting technology with TO—metal band—as well as a comparative analysis of labor productivity. All these aspects constitute further directions of research.

## Figures and Tables

**Table 1:** Material 34 MoCrNi15-φ 40 mm.

No.	Sample Mark	Input Parameters				Average Values			
		*I* [A]	*v_r_* [m/s]	*v_a_* [m/min]	*s* [mm]	*t_t_* [s]	*l_t_* [mm]	*R_a_* [µm]	*TIZ* [mm]
0	1	2	3	4	5	6	7	8	9
1	1	800	20	0.021	0.5	70	2.5	7.7	5.0
2	2′	700	25	0.042	0.5	55	2.2	7.3	5.3
3	3′	950	30	0.063	0.5	44	2.0	8.9	5.5
4	4′	1050	35	0.084	0.5	36	2.0	11.8	5.5
5	5″	700	41	0.105	0.5	30	2.0	19.1	5.5
6	*	1000	20	0.021	1.0	54	2.5	13.1	5.0
7	**	1000	20	0.042	1.0	40	2.5	11.0	5.5
8	***	1050	30	0.063	1.0	36	2.5	9.2	6.0
9	2*	1050	35	0.084	1.0	33	2.0	17.5	6.5
10	3*	1050	41	0.105	1.0	29	2.5	18.2	6.5

**Table 2:** Material RUL-1-Φ 26 MM.

No.	Sample Mark	Input Parameters				Average Values			
		*I* [A]	*v_r_* [m/s]	*v_a_* [m/min]	*s* [mm]	*t_t_* [s]	*l_t_* [mm]	*R_a_* [µm]	*TIZ* [mm]
0	1	2	3	4	5	6	7	8	9
1	1″	320	41	0.021	0.5	44	1.5	15.9	5.0
2	2″	320	41	0.042	0.5	34	1.8	15.1	5.0
3	3″	330	41	0.063	0.5	24	2.1	12.3	4.0
4	3‴	360	41	0.084	0.5	20	2.0	12.7	4.0
5	4″	380	41	0.105	0.5	16	2.0	8.9	3.6
6	1	600	20	0.021	1.0	40	2.3	7.3	5.0
7	5	610	41	0.042	1.0	33	2.5	5.9	5.6
8	2	650	20	0.063	1.0	26	2.3	8.4	6.2
9	3	670	20	0.084	1.0	26	2.5	7.0	7.0
10	7	650	20	0.105	1.0	23	2.5	5.6	7.5

**Table 3:** The dependence of the cutting time, the feed rate of the Processing Object (PO), and the thickness of the metal band.

No.	*s* [mm]	*v_a_* [m/min]	*t_t_* [s]
0	1	2	3
1	0.5	0.021	70
2	0.5	0.042	55
3	0.5	0.063	44
4	0.5	0.084	36
5	0.5	0.105	30
6	1.0	0.021	54
7	1.0	0.042	40
8	1.0	0.063	36
9	1.0	0.084	33
10	1.0	0.105	29

**Table 4:** The dependence of the width of the cut, on the relative speed of the Transfer Object (TO), and on the thickness of the metal band.

No.	*s* [mm]	*v_r_* [m/s]	*l_t_* [mm]
0	1	2	3
1	0.5	20	2.5
2	0.5	25	2.2
3	0.5	30	2.0
4	0.5	35	2.0
5	0.5	41	1.8
6	1.0	20	2.6
7	1.0	20	2.3
8	1.0	30	2.1
9	1.0	35	2.0
10	1.0	41	1.8

**Table 5:** The dependence of the *R_a_*, on the working current, on the thickness of the metal band, and on the *v_a_* of PO.

No.	*I* [A]	*R_a_* [µm]	*v_a_* [m/min]	*s* [mm]
0	1	2	3	4
1	1000	13.5	0.021	1.0
2	950	11.0	0.042	1.0
3	1050	16.7	0.063	1.0
4	1050	17.5	0.084	1.0
5	1050	18.2	0.105	1.0
6	800	7.7	0.021	0.5
7	700	7.3	0.042	0.5
8	950	8.9	0.063	0.5
9	1050	11.8	0.084	0.5
10	700	19.1	0.105	0.5

**Table 6:** Dependence of the thermal influenced area on the working current, on the feed rate of PO, and by the thickness of the metal band.

No.	*v_a_* [m/min]	*I* [A]	*TIZ* [mm]	*s* [mm]
0	1	2	3	4
1	0.021	1000	5.0	1.0
2	0.042	1020	5.6	1.0
3	0.063	1050	6.0	1.0
4	0.084	1050	6.5	1.0
5	0.105	1070	6.5	1.0
6	0.021	500	5.0	0.5
7	0.042	550	5.3	0.5
8	0.063	550	5.5	0.5
9	0.084	650	5.5	0.5
10	0.105	700	5.5	0.5

**Table 7:** The dependence of the cutting time, the feed rate of the PO, and the thickness of the metal band.

No.	*s* [mm]	*v_a_* [m/min]	*t_t_* [s]
0	1	2	3
1	0.5	0.021	44
2	0.5	0.042	34
3	0.5	0.063	24
4	0.5	0.084	20
5	0.5	0.105	16
6	1.0	0.021	40
7	1.0	0.042	33
8	1.0	0.063	28
9	1.0	0.084	26
10	1.0	0.105	23

**Table 8:** The dependence of the width of the cut, on the relative speed of the TO and on the thickness of the metal band.

No.	*s* [m]	*v_r_* [m/s]	*l_t_* [mm]
0	1	2	3
1	0.5	20	2.5
2	0.5	25	2.3
3	0.5	30	2.1
4	0.5	35	2.0
5	0.5	41	1.8
6	1.0	20	2.8
7	1.0	25	2.6
8	1.0	30	2.4
9	1.0	35	2.1
10	1.0	41	1.8

**Table 9:** The dependence of the *R_a_*, on the feed rate of *PO* and on the thickness of the metal band.

No.	*v_a_* [m/min]	*I* [A]	*R_a_* [µm]	*s* [mm]
0	1	2	3	4
1	0.021	600	5.9	1.0
2	0.042	600	7.3	1.0
3	0.063	600	8.7	1.0
4	0.084	610	7.0	1.0
5	0.105	475	6.5	1.0
6	0.021	320	15.9	0.5
7	0.042	320	15.1	0.5
8	0.063	330	13.3	0.5
9	0.084	360	12.7	0.5
10	0.105	380	8.9	0.5

**Table 10:** The dependence of the thermal influenced zone, on the feed rate of PO and on the thickness of the metal band.

No.	*v_a_* [m/min]	*I* [A]	*TIZ* [mm]	*s* [mm]
0	1	2	3	4
1	0.021	600	5.0	1.0
2	0.042	610	5.6	1.0
3	0.063	650	6.2	1.0
4	0.084	670	7.0	1.0
5	0.105	650	7.5	1.0
6	0.021	450	6.0	0.5
7	0.042	500	6.2	0.5
8	0.063	700	7.0	0.5
9	0.084	650	6.8	0.5
10	0.105	475	6.6	0.5
